# Racial/Ethnic Differences in Diabetes Screening and Hyperglycemia Among US Women After Gestational Diabetes

**DOI:** 10.5888/pcd16.190144

**Published:** 2019-10-24

**Authors:** Julie K. Bower, Brittney N. Butler, Seuli Bose-Brill, Jennifer Kue, Christina L. Wassel

**Affiliations:** 1 The Ohio State University College of Public Health, Columbus, Ohio; 2 Kirwan Institute for the Study of Race and Ethnicity, The Ohio State University, Columbus, Ohio; 3 The Ohio State University College of Medicine, Columbus, Ohio; 4 The Ohio State University College of Nursing, Columbus, Ohio; 5 Applied Sciences, Premier, Inc, Charlotte, North Carolina

## Abstract

**Introduction:**

Gestational diabetes mellitus (GDM) is the most common complication of pregnancy and is associated with an increased risk for type 2 diabetes. Racial/ethnic minority populations are at a higher risk than non-Hispanic white populations of developing type 2 diabetes after GDM. The aim of this study was to describe racial/ethnic differences in hyperglycemia and receipt of screening services in a nationally representative sample of women with a history of GDM.

**Methods:**

Our sample included 765 women from the US National Health and Nutrition Examination Survey (2007–2016) with a history of GDM. We used logistic, multinomial, linear, and proportional hazards regression to evaluate racial/ethnic differences in development of diabetes after GDM, hyperglycemia (measured by HbA_1c_), and receipt of diabetes screening services.

**Results:**

Non-Hispanic black women had 63% higher risk and Hispanic women and “other” racial/ethnic women had more than double the risk for diabetes compared with non-Hispanic white women. Among women with a GDM history who did not receive a diagnosis of diabetes by the time of the study examination, both non-Hispanic black women and Hispanic women were more likely than non-Hispanic white women to be in the prediabetes or diabetes range (measured HbA_1c_ ≥5.7%). However, non-Hispanic black women had 2.07 (95% confidence interval, 1.29–3.81) times the odds of being screened for diabetes compared with non-Hispanic white women (*P* = .02).

**Conclusion:**

Delays in identification of hyperglycemia and diagnosis of diabetes in racial/ethnic minority women may reflect differential delivery of guideline-based care or poor follow-up of abnormal screening test results.

**Summary**

**What is already known on this topic?**

Racial/ethnic minority women are disproportionately affected by type 2 diabetes. Among racial/ethnic minority women, non-Hispanic black women are at lowest risk for gestational diabetes mellitus (GDM) but highest risk for conversion to type 2 diabetes after GDM.

**What is added by this report?**

In this nationally representative population of US women with a history of GDM, non-Hispanic black women, compared with other racial/ethnic groups, were most likely to report receiving diabetes screening services in the past 3 years. However, non-Hispanic black and Hispanic women had higher HbA_1c_ levels and were more likely to have measured HbA_1c_ values in the prediabetes or diabetes range.

**What are the implications for public health practice?**

Screening for and prompt diagnosis of type 2 diabetes after GDM is critical in reducing complications. Differential receipt of follow-up services by racial/ethnic minority women may exacerbate disparities in the prevalence of type 2 diabetes.

## Introduction

Gestational diabetes mellitus (GDM) affects 2% to 14% of all pregnancies in the United States and confers a significant and lifelong health challenge for both mother and infant, including an increased risk for type 2 diabetes (1–3). Approximately 60% of women with GDM will develop diabetes within 10 years after delivery, and GDM history is associated with a 7-fold increased lifetime risk for diabetes (2,3). This population comprises a high-risk group that should be targeted for enhanced diabetes screening.

Clinical guidelines recommend postpartum glucose screening 4 to 12 weeks after a GDM pregnancy, yet an inadequate proportion of women with GDM receive these screenings (4). Estimates for receipt of the postpartum glucose screening vary between one-third and three-quarters of postpartum women, depending on the population, with low-income and racial/ethnic minority populations less likely to meet guideline-based screening recommendations (5–7).

For people who do not receive a diagnosis of diabetes at the postpartum visit (90%–95% of all those screened), clinical guidelines recommend enhanced rescreening (8). For people with postpartum glucose screening results in the normoglycemic range, rescreening is recommended at least every 3 years; people with values in the prediabetes range should be rescreened annually. This repeated screening approach improves timely identification of prediabetes or type 2 diabetes and increases success of efforts to prevent or delay progression to type 2 diabetes.

The likelihood of developing type 2 diabetes after GDM differs across racial/ethnic groups. For example, black women with GDM are nearly 10 times as likely as women of all racial/ethnic groups without GDM to develop type 2 diabetes (9). We hypothesized that these observed disparities may partially be driven by differential receipt of screening services. Thus, the aim of this study was to describe racial/ethnic differences in the development of type 2 diabetes, levels of glycated hemoglobin (HbA_1c_) (a measure of hyperglycemia), and receipt of screening services based on clinical guidelines in a nationally representative sample of women with a history of GDM.

## Methods

We analyzed data from the National Health and Nutrition Examination Survey (NHANES) for 2007–2016. NHANES is a cross-sectional, nationally representative survey of the US civilian noninstitutionalized population conducted by the National Center for Health Statistics (10). This study was a secondary data analysis that included 765 NHANES participants with a history of GDM who self-identified as female, were aged 18 years or older, and had self-reported information on current diabetes status at the time of their NHANES examination. A human subjects review board approved data collection procedures, and written informed consent was obtained from all study participants (11).

### Measures

Race/ethnicity and other demographic characteristics, GDM and diabetes diagnoses, current use of insulin, reproductive history, health behaviors, health history, family history, health insurance status, health care access, and receipt of diabetes screening tests were determined by self-report. We categorized race/ethnicity as non-Hispanic white, non-Hispanic black, Hispanic, and “other.” The “other” category comprises women in all other racial/ethnic categories indicated in NHANES as well as women who indicated multiple races/ethnicities. Income-to-poverty ratio was quantified by dividing family income by the poverty threshold determined by the US Department of Health and Human Services, specific to family size; a poverty-income ratio below 1 indicates that the family is below the poverty threshold. GDM history was defined as self-reported diagnosis of diabetes for the first time during pregnancy; age at GDM diagnosis was also reported. Development of type 2 diabetes after GDM was defined at the time of NHANES examination as self-reported diagnosis of diabetes by a health care provider (not including diabetes diagnosed during pregnancy) or use of insulin; age at diabetes diagnosis was also reported by participants. For women who did not develop diabetes after GDM, receipt of screening for diabetes during the previous 3 years was determined by using the following question: “Have you had a blood test for high blood sugar or diabetes within the past three years?” Blood pressure, body mass index (BMI), waist circumference, and lipids were directly measured by using standard methods described previously (12). HbA_1c_ was measured by using high-performance liquid chromatography, standardized to the Diabetes Control and Complications Trial assay (13).

### Statistical analysis

We assessed differences in participant characteristics across racial/ethnic groups by analysis of variance or χ^2^ test as appropriate. For analyses, we defined undiagnosed diabetes as measured HbA_1c_ equal to or greater than 6.5% in the absence of a diagnosis of diabetes or use of insulin and prediabetes as HbA_1c_ from 5.7% to 6.4% in the absence of a diagnosis of diabetes or use of insulin. We used multinomial logistic regression to evaluate the association of race/ethnicity with undiagnosed diabetes (HbA_1c_ ≥6.5%), prediabetes (HbA_1c_ 5.7%–6.4%), and no diabetes (HbA_1c_ <5.7%) at the time of NHANES examination among women with no diabetes (as defined by self-report diagnosis or use of insulin). We evaluated associations of race/ethnicity with HbA_1c_ as a continuous outcome at the time of NHANES examination by using multivariable linear regression, stratified by women with and without diabetes.

Cox proportional hazards regression was used to assess the association of race/ethnicity with development of type 2 diabetes after GDM, using the difference between age at GDM diagnosis and age at diabetes diagnosis after pregnancy (if applicable) as the time to event; for participants who did not develop diabetes, we calculated follow-up time as the difference between GDM diagnosis and current age. Women were excluded if they reported an age at type 2 diabetes diagnosis that was before the age at GDM diagnosis or had missing information on age at type 2 diabetes diagnosis or GDM diagnosis. The proportional hazards assumption was assessed, and it was not violated.

Associations of race/ethnicity, socioeconomic status, and access to health care with receipt of screening services were modeled by using logistic regression among women without diabetes (based on self-report or insulin use information). Analyses incorporated the NHANES sample weights and accounted for the complex sample survey design by using standard methods (12). We used the Taylor series linearization method for variance estimation. Analyses were performed in Stata Statistical Software release 14.2 (StataCorp LLC).

## Results

Among 765 women with a history of GDM, 24.4% developed type 2 diabetes after GDM (weighted percentage). This did vary, although not significantly, across race/ethnicity and was highest among non-Hispanic black women (30.8%) and Hispanic women (31.0%) (Table 1). Approximately one-fifth (22.1%) of non-Hispanic white women and 18.3% of women of “other” race/ethnicity developed type 2 diabetes after GDM (Table 1). Women differed significantly across racial/ethnic groups at the time of NHANES examination in the number of past pregnancies (*P* < .001), BMI (*P* <.001), hypertension prevalence (*P* = .015), and waist circumference (*P* <.001) (Table 1). Hispanic women were most likely to report not having health insurance (34.8% of Hispanic women, compared with 12.5% of non-Hispanic white women) (*P* < .001), and 20.0% of Hispanic women reported not having access to a routine location for health care (*P* = .009 across racial/ethnic groups).

**Table 1:** Characteristics of Women Aged ≥18 Years With a History of Gestational Diabetes Mellitus (N = 765), National Health and Nutrition Examination Survey (NHANES), 2007–2016^a^

Characteristic	Non-Hispanic White (n = 267)	Non-Hispanic Black (n = 138)	Hispanic (n = 266)	Other^b^ (n = 94)	*P* Value^c^
**Age, y**	46.5 (0.9)	44.4 (1.0)	41.8 (0.7)	42.6 (1.6)	<.001
**No. of live births**	2.4 (0.1)	2.7 (0.1)	3.0 (0.1)	2.0 (0.2)	<.001
**No. of pregnancies**	3.3 (0.1)	3.9 (0.2)	3.9 (0.1)	3.0 (0.2)	<.001
**Body mass index, kg/m^2^**	31.2 (0.5)	32.8 (0.9)	31.7 (0.5)	28.1 (1.3)	<.001
**Waist circumference, cm**	102.0 (1.1)	104.4 (1.9)	101.5 (1.1)	93.8 (2.5)	.02
**Current smoker, %**	19.3	19.6	10.8	9.1	<.001
**Systolic blood pressure, mm Hg**	116.5 (1.1)	123.9 (1.7)	120.7 (1.3)	119.5 (4.4)	.23
**Diastolic blood pressure, mm Hg**	70.9 (0.7)	71.9 (1.1)	68.9 (1.0)	69.8 (1.6)	.02
**Hypertension, %**	37.5	56.1	34.1	34.5	.02
**Total cholesterol, mg/dL**	198.8 (3.1)	186.7 (3.6)	200.4 (4.2)	198.8 (10.8)	.52
**High-density lipoprotein cholesterol, mg/dL**	54.3 (1.1)	54.0 (1.5)	52.2 (1.4)	53.4 (2.2)	.40
**Family history of diabetes, %**	58.2	69.8	59.7	61.7	.72
**Developed type 2 diabetes after GDM, %**	22.1	30.8	31.0	18.3	.01
**HbA_1c_ category, % **					
No current diabetes, <5.7%	53.2	32.5	41.1	53.2	.11
No current diabetes, 5.7%–6.4%	20.2	28.5	21.2	26.5	
No current diabetes, ≥6.5%	4.5	8.2	6.7	2.0	
Current diabetes, <7.0%	13.7	13.6	12.4	13.0	
Current diabetes, ≥7.0%	8.4	17.2	18.6	5.4	
**Had diabetes screening in past 3 years^d^**	67.1	77.3	59.2	72.6	.11
**No health insurance, %**	12.5	14.6	34.8	17.0	<.001
**No routine health care location, %**	9.2	3.4	20.0	12.2	.009
**Education, %**					
<High school diploma	12.2	16.6	45.3	5.3	<.001
High school diploma or equivalent	20.0	19.4	19.3	15.4	
Some college	39.4	40.6	26.7	27.9	
College graduate or higher	28.3	23.4	8.8	51.4	
**Income-to-poverty ratio^e^**	3.1 (0.1)	2.3 (0.2)	1.8 (0.1)	3.4 (0.2)	<.001

Abbreviations: HbA_1c_, glycated hemoglobin A_1c_.

a All values are weighted mean (standard error) for continuous variables and weighted percentage for categorical or binary variables, unless otherwise indicated. Unweighted sample size ranged from 630–765 because of missing data.

b Comprises women in all other racial/ethnic categories indicated in NHANES as well as women who indicated multiple race/ethnicities.

c Analysis of variance used to determine *P* values for continuous values and χ^2^ tests for categorical values.

d Calculated only among those without diabetes at the time of NHANES data collection (unweighted n = 534).

e Income-to-poverty ratio was quantified by dividing family income by the poverty threshold determined by the US Department of Health and Human Services, specific to family size; a poverty-income ratio below 1 indicates that the family is below the poverty threshold.

### Associations of race/ethnicity with HbA_1c_

In a fully adjusted model (Table 2), non-Hispanic black women, Hispanic women, and women in “other” racial/ethnic groups had significantly higher HbA_1c_ levels than did non-Hispanic white women. Among women with diabetes (by self-report or insulin use), non-Hispanic black and Hispanic women had 1.32% (*P* = .002) and 1.31% (*P* < .001) higher HbA_1c_, respectively, compared with non-Hispanic white women; the “other” racial/ethnic group did not differ significantly from the non-Hispanic white group (*P* = .57). Among women with no diabetes, non-Hispanic black and Hispanic women had 0.34% higher HbA_1c_ (*P* = .004 among non-Hispanic black women; *P* = .001 among Hispanic women), whereas the “other” racial/ethnic group had a 0.14% higher HbA_1c_ (*P* = .03), compared with non-Hispanic white women. In fully adjusted models (Table 2), the relative risk ratio for prediabetes was 3.4-fold higher (95% confidence interval [CI], 1.60–7.04); *P* = .002) and for HbA_1c_ ≥6.5% was 5.2-fold higher (95% CI, 1.39–19.70); *P* = .02) among non-Hispanic black women than among non-Hispanic white women. Among Hispanic women, the relative risk ratio for prediabetes was 2.2-fold higher (95% CI, 1.17–4.17; *P* = .02) and for HbA_1c_ ≥6.5% was 6.7-fold higher (95% CI, 2.51–17.98; *P* <.001) than among non-Hispanic white women.

**Table 2:** Racial/Ethnic Differences Among Women Aged ≥18 Years in Association With HbA_1c_, National Health and Nutrition Examination Survey (NHANES), 2007–2016

Characteristic	RRR (95% CI) [*P*]		HbA_1c_, β (95% CI) [*P*]	
	HbA_1c_ 5.7%–6.4%^a^ (Unweighted n = 534)	HbA_1c_ ≥6.5%^a^ (Unweighted n = 534)	No Diabetes (Unweighted n = 534)	Diabetes (Unweighted n = 231)
**Model 1: Unadjusted**				
Non-Hispanic white	1.00 [Reference]	1.00 [Reference]	1.00 [Reference]	1.00 [Reference]
Non-Hispanic black	2.31 (1.30 to 4.13) [.005]	2.98 (1.01 to 8.79) [.05]	0.31 (0.07 to 0.56) [.01]	1.12 (0.19 to 2.05) [.02]
Hispanic	1.36 (0.82 to 2.26) [.22]	1.94 (0.80 to 4.68) [.14]	0.24 (0.05 to 0.43) [.01]	0.90 (0.26 to 1.54) [.006]
Other	1.32 (0.49 to 3.54) [.58]	0.44 (0.11 to 1.86) [.26]	0.06 (−0.10 to 0.21) [.45]	−0.30 (−0.83 to 0.23) [.26]
**Model 2: Adjusted for age, education, marital status, and health insurance status**				
Non-Hispanic white	1.00 [Reference]	1.00 [Reference]	1.00 [Reference]	1.00 [Reference]
Non-Hispanic black	3.46 (1.77 to 6.78) [<.001]	4.94 (1.53 to 15.96) [.008]	0.40 (0.15 to 0.65) [.002]	1.30 (0.46 to 2.14) [.003]
Hispanic	1.98 (1.07 to 3.67) [.03]	3.49 (1.57 to 8.04) [.004]	0.34 (0.14 to 0.54) [.001]	1.17 (0.55 to 1.78) [<.001]
Other	1.97 (0.70 to 5.50) [.19]	0.61 (0.12 to 3.23) [.56]	0.14 (−0.01 to 0.29) [.06]	−0.29 (−0.84 to 0.27) [.31]
**Model 3: Model 2 + body mass index, waist circumference, hypertension status, and high-density lipoprotein cholesterol**				
Non-Hispanic white	1.00 [Reference]	1.00 [Reference]	1.00 [Reference]	1.00 [Reference]
Non-Hispanic black	3.35 (1.60 to 7.04) [.002]	5.24 (1.39 to 19.70) [.02]	0.34 (0.11 to 0.57) [.004]	1.32 (0.48 to 2.15) [.002]
Hispanic	2.20 (1.17 to 4.17) [.02]	6.72 (2.51 to 17.98) [<.001]	0.34 (0.15 to 0.53) [.001]	1.31 (0.71 to 1.91); [<.001]
Other	2.09 (0.83 to 5.30) [.12]	0.96 (0.08 to 11.18) [.97]	0.14 (0.01 to 0.27) [.03]	−0.15 (−0.67 to 0.37) [.57]

Abbreviations: CI, confidence interval; HbA_1c_, glycated hemoglobin A_1c_; RRR, relative risk ratio.

a Among women aged ≥18 years who had complete information on covariates and who did not self-report a diagnosis of diabetes by a health care provider or insulin use at the time of the NHANES examination. Multinomial regression models were used to assess undiagnosed diabetes (HbA_1c_ ≥ 6.5%), prediabetes (HbA_1c_, 5.7%–6.4%), and no diabetes (HbA_1c _< 5.7%). No diabetes is reference group (or base outcome).

### Associations of race/ethnicity with development of type 2 diabetes after GDM

Among the 765 women with a history of GDM, 6 were missing information on age at diabetes diagnosis and were excluded from analysis. In fully adjusted models, non-Hispanic black women had a 63% higher risk (95% CI, 1.11–2.39; *P* = .01), Hispanic women more than double the risk (hazard ratio = 2.22; 95% CI, 1.47–3.35; *P* < .001), and “other” racial/ethnic women approximately double the risk (hazard ratio = 2.08; 95% CI, 1.01–4.28; *P* = .047) of developing type 2 diabetes following GDM compared with non-Hispanic whites (Table 3).

**Table 3:** Racial/Ethnic Differences in the Development of Type 2 Diabetes Among Women Aged ≥18 Years With a History of Gestational Diabetes Mellitus (N = 759), National Health and Nutrition Examination Survey, 2007–2016^a^

Model	Non-Hispanic White	Hazard Ratio (95% Confidence Interval) [*P *Value]		
		Non-Hispanic Black	Hispanic	Other
Model 1^b^	1.00 [Reference]	1.95 (1.32–2.88) [.001]	2.17 (1.44–3.25) [<.001]	1.18 (0.56–2.51) [.66]
Model 2^c^	1.00 [Reference]	1.71 (1.13–2.58) [.01]	1.68 (1.09–2.60) [.02]	1.57 (0.80–3.09) [.19]
Model 3^d^	1.00 [Reference]	1.63 (1.11–2.39) [.01]	2.22 (1.47–3.35) [<.001]	2.08 (1.01–4.28) [.047]

a Of 765 women aged ≥18 years with a history of gestational diabetes mellitus, 6 did not have data on age at diabetes diagnosis.

b Unadjusted.

c Adjusted for age, education, marital status, and health insurance status.

d Model 2 + body mass index, waist circumference, hypertension status, and high-density lipoprotein cholesterol.

### Receipt of screening among women without diabetes

Among women without diabetes (defined by self-report or insulin use) at the NHANES examination, 67.1% reported having had a diabetes screening test at least once in the past 3 years. By race/ethnicity, this percentage was 67.1% among non-Hispanic white women, 77.3% among non-Hispanic black women, 59.2% among Hispanic women, and 72.6% among women in “other” racial/ethnic groups (Table 1). In adjusted models, non-Hispanic black women had 2.07 times the odds of being screened for diabetes compared with non-Hispanic white women (95% CI, 1.29–3.81; *P* = .02) (Table 4). Women with a higher income-to-poverty ratio (odds ratio [OR] per 1-unit increment = 1.27; 95% CI, 1.08–1.49; *P* = .005) were more likely to have been screened, and women who reported no access to a routine place for health care (compared with those with access to ≥1 location for regular health care) had 0.44 times the odds of being screened (95% CI, 0.22–0.90; *P* =.02) (Figure). Other potential factors were considered but were removed from the final model because of no observed association; these factors were education, marital status, current age, and health insurance status. Sensitivity analyses adjusting for clinical diabetes risk factors only slightly attenuated the findings on race/ethnicity.

**Table 4:** Racial/Ethnic Differences in Screening for Diabetes Among Women Aged ≥18 Years Without Current Diabetes (n = 496), National Health and Nutrition Examination Survey, 2007–2016^a^

Model	Non-Hispanic White	Odds Ratio (95% Confidence Interval) [*P* Value]		
		Non-Hispanic Black	Hispanic	Other
Unadjusted	1.00 [Reference]	1.89 (0.98–3.22) [.06]	0.66 (0.40–1.07) [.09]	1.16 (0.55–2.48) [.69]
+ Income-to-poverty ratio^b^	1.00 [Reference]	2.22 (1.22–4.03) [.009]	0.91 (0.53–1.56) [.73]	1.11 (0.52–2.35) [.78]
+ Routine health care location	1.00 [Reference]	2.07 (1.29–3.81) [.02]	1.01 (0.58–1.78) [.96]	1.10 (0.51–2.38) [.24]

a Among 534 women aged ≥18 years with no diabetes, 38 were missing information on income-to-poverty ratio and routine health care location.

b Income-to-poverty ratio was quantified by dividing family income by the poverty threshold determined by the US Department of Health and Human Services, specific to family size; a poverty-income ratio below 1 indicates that the family is below the poverty threshold.

**Figure:**
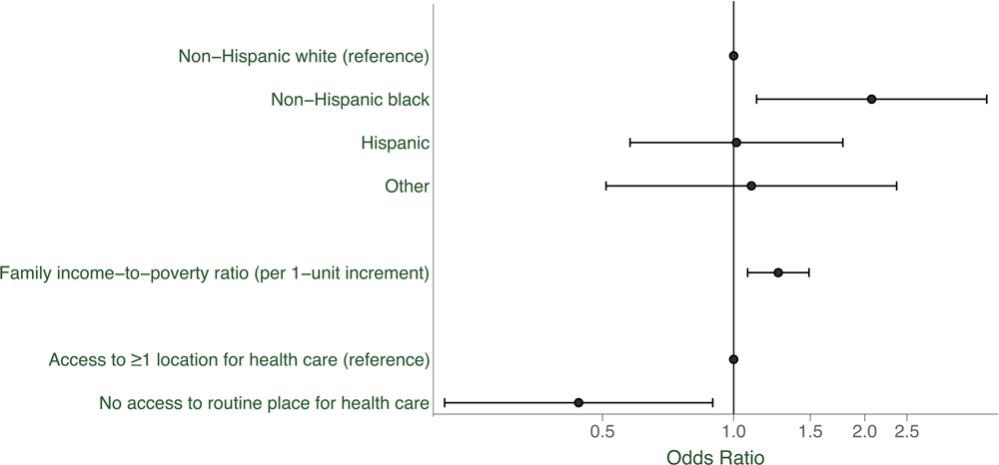
Racial/ethnic differences in having been screened for diabetes at least once in the past 3 years, US women without diabetes (n = 496). Odds ratios were adjusted for all other variables in the figure. Error bars indicate 95% confidence intervals.

**Table:** 

Characteristic	Odds Ratio (95% Confidence Interval)
Non-Hispanic white	1 [Reference]
Non-Hispanic black	2.07 (1.13–3.81)
Hispanic	1.01 (0.58–1.78)
Other	1.10 (0.51–2.37)
Family income-to-poverty ratio (per 1-unit increment)	1.27 (1.08–1.49)
Access to ≥1 location for health care	1 [Reference]
No access to routine place for health care	0.44 (0.22–0.90)

## Discussion

In our study, non-Hispanic black and Hispanic women were at higher risk for developing type 2 diabetes after GDM compared with non-Hispanic white women, and they had higher HbA_1c_ levels. Among all racial/ethnic groups examined, non-Hispanic black women were most likely to have measured HbA_1c_ values in the prediabetes (HbA_1c_ 5.7%–6.4%) and diabetes range (HbA_1c_ ≥ 6.5% in the absence of a diagnosis of diabetes). Both groups had higher rates of prediabetes and diabetes compared with non-Hispanic white women. Non-Hispanic black and Hispanic women had significantly higher mean HbA_1c_ levels, regardless of current diagnosed diabetes status.

Racial/ethnic disparities in risk for type 2 diabetes after GDM are well documented. Although non-Hispanic white and black women are at similar risk for GDM, black women have more than 2 times higher risk for developing type 2 diabetes after GDM compared with non-Hispanic white women (9). Heterogeneity in the burden of GDM risk and consequences within these subgroups cannot be fully explained by biological factors and traditional diabetes risk factors, and it is likely strongly influenced by social determinants and differential patterns of clinical practice (14). In our study, racial/ethnic differences in risk for diagnosed type 2 diabetes after GDM persisted even after adjustment for traditional diabetes risk factors such as age, education level, and BMI, and health insurance status. These findings demonstrate a gap in our understanding of individual and clinical practice factors — beyond traditional diabetes risk factors — influencing development of type 2 diabetes among racial/ethnic minority women.

The proportion of women reporting receipt of a diabetes screening test varied by race/ethnicity in the US population of women with a GDM history, despite the clinical recommendation that all women with GDM history be screened every 1 to 3 years. The finding that non-Hispanic black women without current diabetes, but with a history of GDM, were more likely to be screened for diabetes is consistent with their more adverse diabetes risk profile, including higher mean blood pressure, BMI, and prevalence of family history of diabetes. Despite being screened and demonstrating a higher prevalence of hyperglycemia, they were less likely to receive a diagnosis of diabetes. In unadjusted (crude) models, non-Hispanic black women had higher odds and Hispanic women had lower odds of being screened compared with non-Hispanic white women. This association became nonsignificant after adjustment for sociodemographic, clinical, and health care access factors for Hispanic women, but the association became stronger for non-Hispanic black women.

The lower percentage of Hispanic women who reported being screened for diabetes is consistent with the observed lower percentage who had current health insurance and lower percentage reporting access to a routine location to obtain health care compared with non-Hispanic white women. More than 30% of Hispanic women with a GDM history were subsequently diagnosed with diabetes, higher than the percentage among non-Hispanic white women and similar to the percentage among non-Hispanic black women. However, among women that had not been diagnosed with diabetes, Hispanic women had lower odds of being screened for diabetes in the previous 3 years compared with all other racial/ethnic groups in unadjusted models. The association of race/ethnicity with receipt of screening became nonsignificant for Hispanic women after adjusting for measures of household income and access to a routine health care location. This finding suggests that access to screening services may play an important role in the observed lower likelihood of diabetes screening in this population.

Our findings also support research that non-Hispanic black women, in particular, are more likely to have undiagnosed prediabetes or diabetes and have a higher prevalence of suboptimal HbA_1c_ (15–19). These findings might partially reflect delays in early diagnosis of diabetes that may result from differential delivery of guideline-based care and/or follow-up of abnormal screening test results. Non-Hispanic black women may be screened more frequently because of perceived increased risk or higher prevalence of known diabetes risk factors, but evidence suggests that we are missing opportunities for early identification of hyperglycemia in this population. Health care providers play an important role in the diagnosis and treatment of diabetes for at-risk populations. Timely coordinated medical care is crucial because racial/ethnic minority patients are often diagnosed at later stages of disease than are other racial/ethnic groups (20). Studies are needed to further examine racial/ethnic differences in diagnosis and treatment of diabetes, as well as the underlying factors that may limit adherence to guideline-based care among racial/ethnic minority populations.

Although NHANES is a cross-sectional survey, it collects detailed information about pregnancy history and timing of diabetes diagnoses, allowing for estimation of diabetes risk after GDM. Additionally, non-Hispanic black and Hispanic women were oversampled to provide a large study population to evaluate racial/ethnic differences; Asian women were not oversampled in most survey years and, therefore, we were not able to look at this subpopulation separately. Finally, diabetes diagnosis information was collected via self-report; potential misclassification cannot be ruled out. However, the study is strengthened by inclusion of a nationally representative sample of US women with a GDM history and extensive information about their social and family history, health behaviors, health status and history, and several indicators related to access to health services.

Screening and diagnosis of GDM is critical in reducing complications in women and their infants, as well as preventing onset of type 2 diabetes. Differential receipt of follow-up services by racial/ethnic minority women may exacerbate observed disparities in the burden of type 2 diabetes. Our study showed racial/ethnic disparities in diabetes diagnosis, receipt of diabetes screening tests, and HbA_1c_ levels. Additional investigation to identify underlying factors contributing to this observed disparity will be particularly important to inform recommendations to ensure more equitable delivery of quality care across populations.
